# Assessing Plantar Skin Epidermal Layer in Response to Various Insole Hardness via Deep Learning Enabled Optical Coherence Tomography Analysis

**DOI:** 10.1002/jbio.70330

**Published:** 2026-08-04

**Authors:** Ardha Ardea Prisilla, Yih‐Kuen Jan, Ben‐Yi Liau, Chien‐Cheng Tai, Gilang Titah Ramadhan, Sheena Christabel Pravin, Kiruthika Venkataramani, Winson Chiu‐Chun Lee, Congo Tak Shing Ching, Chi‐Wen Lung

**Affiliations:** ^1^ Department of Fashion Design Sekolah Tinggi Desain LaSalle Jakarta Jakarta Indonesia; ^2^ Department of Digital Media Design Asia University Taichung Taiwan; ^3^ Rehabilitation Engineering Lab, Department of Health and Kinesiology University of Illinois at Urbana‐Champaign Urbana Illinois USA; ^4^ Department of Automatic Control Engineering Feng Chia University Taichung Taiwan; ^5^ School of Public Health, College of Public Health Taipei Medical University New Taipei City Taiwan; ^6^ Departement of Informatics Tiga Serangkai University Surakarta Indonesia; ^7^ School of Electronics Engineering Vellore Institute of Technology Chennai India; ^8^ School of Mechanical, Materials, Mechatronics and Biomedical Engineering University of Wollongong Wollongong Australia; ^9^ Graduate Institute of Biomedical Engineering National Chung Hsing University Taichung Taiwan; ^10^ Department of Creative Product Design Asia University Taichung Taiwan; ^11^ Department of Medical Research China Medical University Hospital Taichung Taiwan

**Keywords:** diabetic foot ulcers, epidermis, insole stiffness, plantar thickness, stratum corneum, walking duration

## Abstract

We tested walking interventions using adjustable air‐insoles with hardness values of 80, 160, and 240 mmHg over walking durations of 10 and 20 min. Optical coherence tomography (OCT) was used to measure the thickness of the stratum corneum (SC) and living epidermis (ED) in three different locations: the big toe (T1), first metatarsal head (M1), and second metatarsal head (M2), and deep learning was used to compare the difference in thickness between SC and ED. The results indicate that SC thickness increases in M1 is statistically significant after 20 min walking interventions using 80 mmHg insole hardness, and SC thickness decreases in T1 and M1 are statistically significant after 20 min walking using 160 mmHg insole hardness. Changes in SC and ED thickness observed in this study highlight their potential relevance in evaluating plantar tissue health in the context of DFU prevention.

## Graphical abstract

The changes in the plantar stratum corneum (SC) and the living epidermis (ED) at the big toe (T1), first metatarsal head (M1), and second metatarsal head (M2) were investigated after a controlled walking experiment on a treadmill at 3.6 mph using 80, 160, and 240 mmHg insole hardness for 10 and 20 min. The thickness changes in SC and ED layers are observed using optical coherence tomography, segmented and analyzed using a deep learning model.

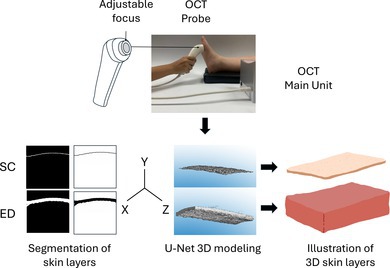

## 1 Introduction

Diabetes Mellitus (DM) is a multifactorial disease that affects most body tissues and is characterized by elevated blood glucose levels above a certain threshold [1]. It is a major public health concern worldwide and is associated with high mortality, morbidity, and disability rates [2]. Diabetes prevalence reached 537 million adults aged 20 to 79 in 2021, and it is estimated to reach 643 million by 2030, according to the International Diabetes Federation [3]. DM expenditure is expected to increase from US$966 billion in 2021 to US$1054 billion by 2045 [2]. As DM can negatively impact health and social insurance systems, policymakers must promptly address this issue [4].

Exercise is a preventive intervention that may benefit patients with diabetes and reduce chronic complications [5, 6]. Walking is the most commonly recommended exercise method for individuals with diabetes [7, 8]. According to several studies, there is a significant increase in the ratio of metabolic, myogenic, respiratory, and cardiac wavelet amplitudes after walking for 10 min at 9 km/h and 20 min at 6 km/h, which shows that walking can help diabetic patients regulate their blood sugar levels [6, 9, 10, 11]. Nevertheless, individuals with DM are more likely to develop ulcers when walking because they generate repeated high pressure and mechanical stress on their feet [12, 13].

Sustained high plantar pressure and mechanical stress can trigger skin breakdown, which can increase the risk of diabetic foot ulcers (DFU) [13]. As DFU develops, the skin undergoes a complex healing process involving three phases: inflammation, proliferation, and remodeling. Immune cells migrate to the wound during the inflammatory phase to combat infection and remove debris, resulting in symptoms such as redness, warmth, swelling, and pain [13, 14]. The inflammatory phase is followed by the proliferative phase, during which new tissue and blood vessels are formed, and fibroblasts produce collagen and other components of the extracellular matrix [15]. Finally, collagen fibers are reorganized, resulting in a wound tissue that is generally thicker and less flexible, especially in the epidermal layers.

In individuals with DFU, walking can compromise epidermal layers that are less protected from external threats, leading to wound development and hindering healing [16]. Therefore, walking with optimal hardness insoles distributes pressure away from high‐stress areas of the foot, particularly around bony prominences, and allows the forefoot to store and reuse mechanical energy during push‐off [17, 18, 19]. Furthermore, owing to repetitive gait movement, the epidermal layers are the first to experience mechanical stress. The epidermal layer of the plantar serves as a shield against external threats, such as pathogens and chemicals, and varies in thickness in different areas of the body [20].

Therefore, changes in epidermal thickness can serve as early indicators of abnormal pressure or friction related to DFU. The stratum corneum (SC) and living epidermis (ED) (e.g., stratum basale and spinosum) serve as the first line of defense, and any reduction in their thickness or structural integrity increases their vulnerability to mechanical stress and microbial invasion [21]. SC is crucial for maintaining this barrier and retaining skin hydration [16]. Impaired SC thickness may indicate poor skin barrier function, leading to an increased risk of skin breakdown and infection, which are key factors in the development of DFU. At the same time, ED is responsible for cell regeneration and wound healing, triggering the activation and migration of Langerhans cells from the SC and ED layers to lymph nodes [22]. In DFU, impaired immune function and Langerhans cell dysfunction can delay wound healing.

However, measuring the thickness of the SC and ED layers remains challenging in clinical practice because of the difficulty in obtaining accurate, reliable, and noninvasive assessments of these parameters [23]. Traditional methods, such as ultrasound or palpation, often either lack precision or require invasive techniques, making it difficult to evaluate SC and ED thickness without compromising skin integrity or patient comfort [24, 25]. Optical coherence tomography (OCT) is emerging as a promising tool to address these limitations [26]. OCT provides high‐resolution, cross‐sectional images of the skin layers, allowing for detailed visualization and quantification of SC and ED thickness. OCT's ability to capture microstructural changes also enables clinicians to detect variations in SC and ED thickness that are associated with early indications of tissue breakdown, which is crucial for understanding skin resilience, especially in high‐risk areas such as the feet of diabetic patients [20, 27].

Because increased mechanical stress at the SC and ED tissue is associated with plantar breakdown, this study hypothesizes that changes in SC and ED thickness can serve as useful indicators for evaluating the effects of insole hardness during walking, and that OCT can detect pressure‐related epidermal changes earlier than conventional methods. Furthermore, the walking duration can influence and be used to monitor changes in the thickness of the SC and ED layers, revealing how these skin layers respond to mechanical stress over time. Although OCT has commonly been used to evaluate epidermal thickness and skin structure, research examining changes in plantar SC and deeper epidermal layers following walking intervention is still relatively limited. This study in healthy participants provides a foundation for selecting appropriate insoles to promote safer and more effective walking and exercise in people with DFU, based on understanding the effect of walking intensities on SC and ED thickness changes. Accordingly, we propose that detecting changes in SC and ED thickness may support timely interventions to reduce the risk of DFU.

## 2 Materials and Methods

### 2.1 Participants

**FIGURE 1:**
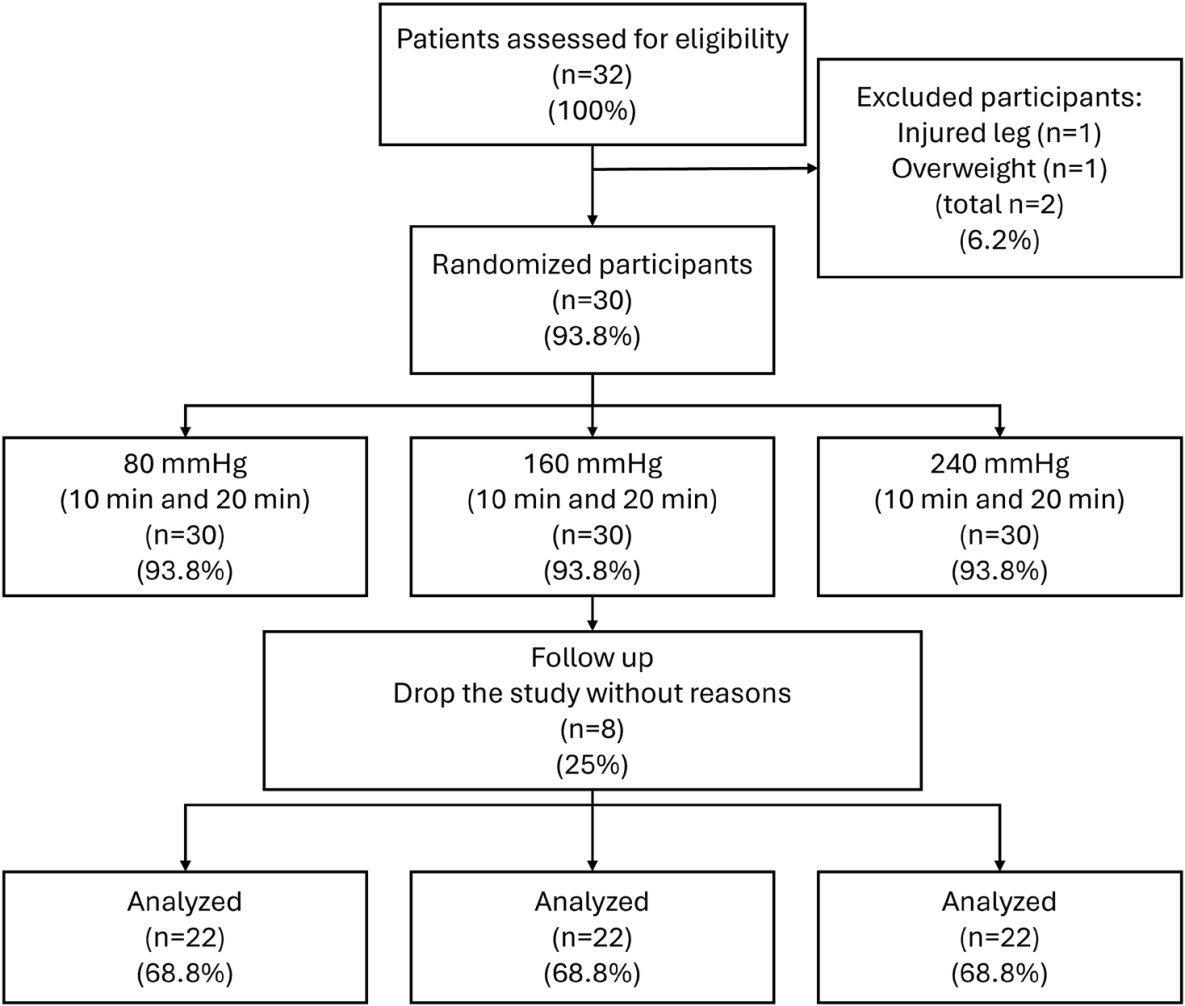
CONSORT diagram showing participant allocation. CONSORT, Consolidated Standards of Reporting Trials.

A total of 32 healthy participants who could walk independently without any device were recruited in the study (14 men and 18 women) between the ages of 21 and 59 years. Study participants were excluded if they had active ulcers on their feet, DM, pain in any lower extremity joint, or a history of foot amputation or other lower extremity surgeries. There were several criteria for selecting the participants, including shoe size (EU size 41–43) for men, shoe size (EU size 36–38) for women, and body weight of less than 80 kg with a dominant right leg. The characteristics of the participants (mean ± SD) were age 27.6 ± 9.6 years, body weight 60.5 ± 13.5 kg, body height 165.1 ± 8.4 cm, and Body Mass Index 22.1 ± 4.6 kg/m^2^. The studies involving human participants were approved by the Central Regional Research Ethics Committee of China Medical University, Taichung, Taiwan (CRREC‐112‐130), and were subsequently registered in the International Trial Registry since 2024/12/24 [ClinicalTrials.gov: Identifier NCT06746597 (https://clinicaltrials.gov/study/NCT06746597)]. This study was conducted in accordance with local legislation and institutional requirements. All participants provided written informed consent to participate in this study and were assured that their personal information would remain confidential. As required by the Declaration of Helsinki, respondents were able to withdraw from the study at any time, and their responses were anonymized. The recruitment period for the participants began in June 2024 and ended in April 2025. From the total 32 participants, 2 participants were excluded from further randomization at the earlier stage, and 8 participants withdrew from the experiment before they completed the 6 walking conditions. Therefore, only the completed data from 22 participants were analyzed. The Consolidated Standards of Reporting Trials (CONSORT) diagram showing participant allocation is presented in Figure 1.

### 2.2 Equipment

In our previous study, we selected three insole hardness values to ensure appropriate elasticity for walking, with the insoles set at three different hardness values (80, 160, and 240 mmHg) [5, 11]. The insoles were composed of thermoplastic polyurethane (Hsin He Hsin Co. Ltd., Taichung, Taiwan). Air insole hardness values were determined using a GS‐701N Shore durometer (Teclock Co. Ltd., Nagano, Japan) [28]. The present study measured three different pressures: an 80 mmHg hardness value at 51.7 ± 1.5 Shore, a 160 mmHg hardness value at 54.7 ± 0.6 Shore, and a 240 mmHg hardness value at 57.7 ± 0.6 Shore [5]. The participants were requested to wear commercial footwear and walk on a treadmill (Cybex DE‐20427 A, Cybex, Taoyuan, Taiwan).

### 2.3 Experimental Procedure

This study used a repeated‐measures randomized crossover design in which all participants completed all six walking conditions (within‐subject) in a random combination of insole hardness and walking duration. The insoles were positioned in the metatarsal and toe regions of footwear. Before walking, the participants removed their socks and shoes and rested in a supine position for 30 min to reduce the influence of previous weight‐bearing activity and muscle fatigue on plantar pressure.

Three different insole hardness values (80, 160, and 240 mmHg) and two walking durations (10 and 20 min) were tested, resulting in six walking conditions. The six walking conditions were completed across a minimum of 3 days (two sessions per day) or a maximum of 6 days (one session per day). For participants completing two sessions in 1 day, a 20‐min recovery period was provided between sessions to reduce potential fatigue and carryover effects. This rest duration was adapted from previous findings [29] showing that 20 min was sufficient for neuromuscular recovery after moderate exercise.

– 80 mmHg for 10 min
– 80 mmHg for 20 min
– 160 mmHg for 10 min
– 160 mmHg for 20 min
– 240 mmHg for 10 min
– 240 mmHg for 20 min

A walking speed of 3.6 mph was used, following the recommendations of the American Guidelines and the American Diabetes Association and previous clinical exercise guidelines, which identify this pace as a safe and commonly prescribed walking speed for individuals with DM [30, 31]. This speed falls within the moderate‐intensity range, making it both realistic and clinically relevant for evaluating the mechanical stress experienced by the SC and ED tissues during daily activities [30]. This study was conducted as part of a broader investigation into the effects of varying insole hardness and walking duration on the biomechanical properties of plantar soft tissue [5].

**FIGURE 2:**
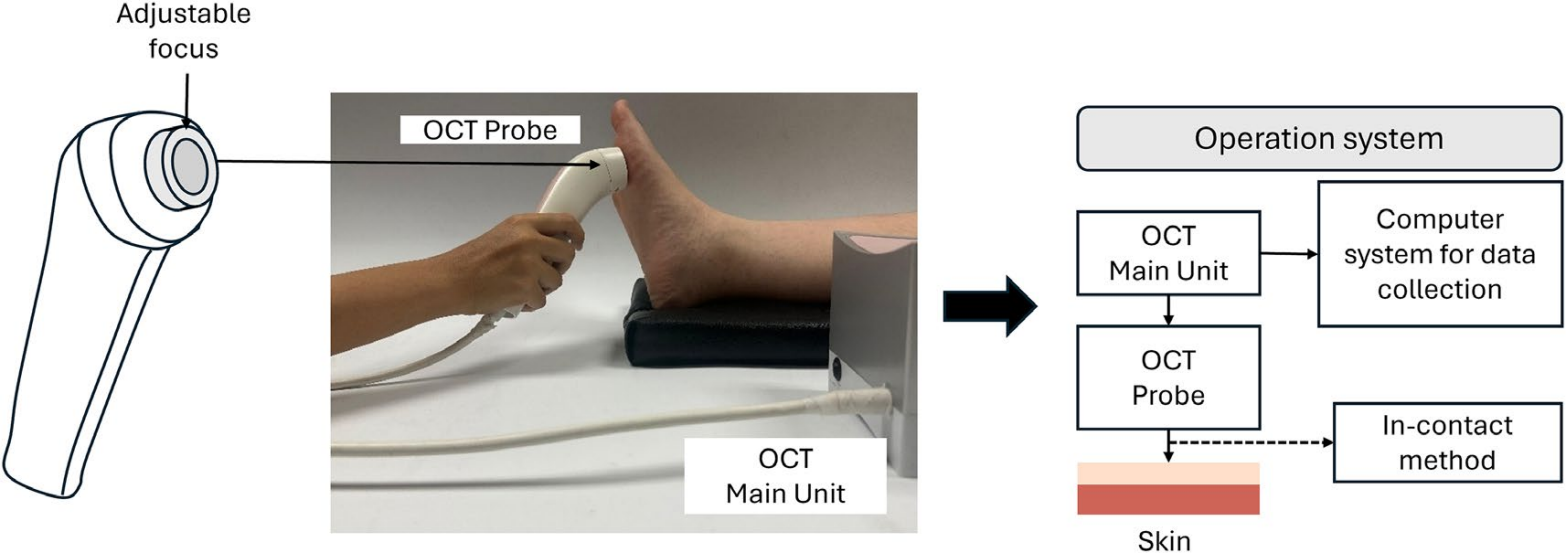
Data Collection using OCT. OCT, optical coherence tomography.

We collected plantar data with OCT before and after walking intervention in three different locations: the big toe (T1), the first metatarsal head (M1), and the second metatarsal head (M2). The anatomical locations corresponding to the T1, M1, and M2 were identified by the same investigator throughout all data collection sessions. To improve measurement consistency, the OCT probe placement was standardized using skin markers at the center of T1, M1, and M2. During scanning, the OCT probes directly touched the skin in a perpendicular direction, and the subject was required to remain still for a maximum of 50 s to prevent motion artifacts from negatively impacting the results of the scanning process (Figure 2). However, slight variations in manual probe positioning may still have occurred between sessions.

The SC and ED layers were identified based on OCT skin characteristics described in a previous study [26]. In healthy skin, the SC generally appeared as the outermost bright layer, while the ED appeared as a darker layer beneath it with a visible boundary above the dermis. To maintain consistency, all manual annotations and validations were performed by the same investigator throughout the study. In addition, the segmentation results were reviewed and discussed with an experienced clinician from Asia University Hospital, who had worked in the field for over 15 years, to support the anatomical interpretation of the layers.

**FIGURE 3:**
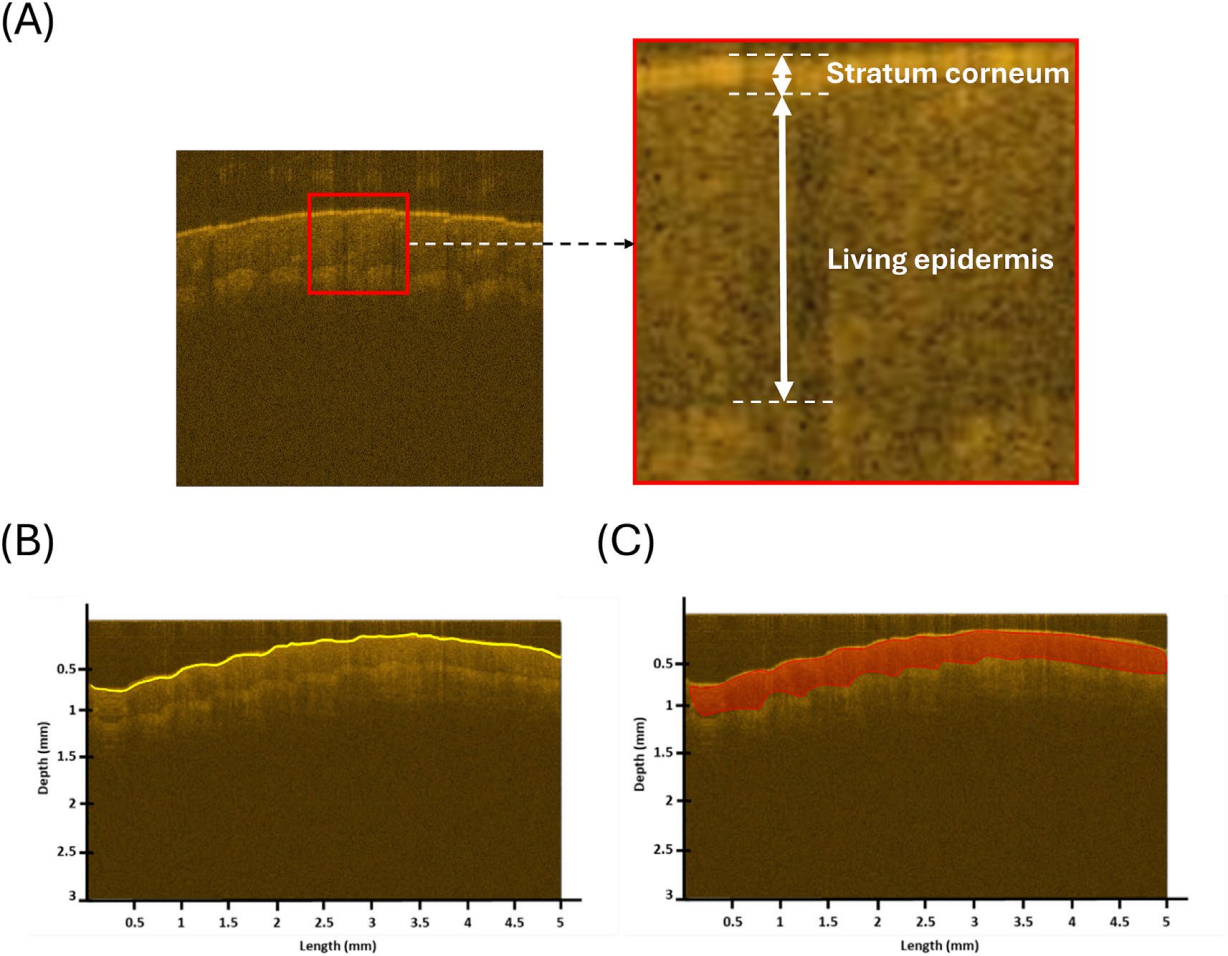
Depth structure of the SC and ED layers. (A) Skin layers, (B) SC image segmentation in yellow, (C) ED image segmentation in red. CT, optical coherence tomography; ED, living epidermis; SC, stratum corneum.

OCT was used to scan and evaluate the properties of the skin layer. The OCT (model OPXSV1‐02F, OPXION Technology Inc., New Taipei City, Taiwan) includes a B‐scan rate of 25 fps, vertical resolution of 7.5 μm, horizontal resolution of 10 μm, and a laptop to run OCT software for image capture. These parameters enable high‐resolution visualization of microstructural changes in skin thickness, surpassing the spatial resolution typically achievable with high‐frequency ultrasound, which is limited to ~30–60 μm axial resolution [32]. The processing speed and frame rate were measured using an imaging system (V1.0, OPXION Technology Inc., New Taipei City, Taiwan). Specifically, the dataset consisted of 500 high‐resolution OCT images acquired for each subject over a period. Each of the images had a pixel size of 1029 mm × 1005 mm × 3 mm, which represents an area of approximately 5 mm × 5 mm, and the frame thickness was 0.01 mm (Figure 3).

### 2.4 U‐Net Segmentation

In medical imaging, the U‐Net is a convolutional neural network (CNN) architecture specifically designed for image segmentation, such as the need for precision localization with limited training data [33]. The U‐Net approach saves time and effort during image analysis by automating segmentation, which would otherwise require manual labeling. Recent advances in deep learning for OCT skin segmentation, particularly with U‐Net, have shown considerable effectiveness and potential in clinical point‐of‐care applications. Liu et al. applied U‐Net to real‐time skin layer segmentation, achieving high precision in delineating the epidermis and detecting lesions [33]. Lin et al. introduced an enhanced convolutional neural network with a graph search (CNN‐GS) to measure epidermal thickness in OCT images rapidly [26].

In this study, we used a dataset consisting of 700 OCT images and 100 epochs, divided into three groups: 60% training, 20% testing, and 20% validation. The segmentation model was developed using a mixed OCT dataset consisting of approximately 30% plantar images from the current study and additional OCT images from other skin regions, including the palm and other anatomical areas. This approach was used to improve model robustness and familiarize the network with SC and ED features across different skin structures. This study was conducted on a machine with the following specifications: Core (TM) i7‐10 700 CPU Intel(R) and an NVIDIA GeForce RTX 3080 GPU. In our previous study, training the U‐Net model with 200 OCT images and 100 epochs produced a high segmentation accuracy, with the SC layer achieving 92.75% and the ED layer 95.42% [34]. These results highlight the effectiveness of U‐Net in performing detailed segmentation tasks on complex medical image data. These results are comparable to, and in some cases higher than, those reported in previous OCT segmentation studies, where accuracies typically ranged between 85% and 96% [35, 36]. This indicates that our model is sufficiently robust for quantifying epidermal thickness.

**FIGURE 4:**
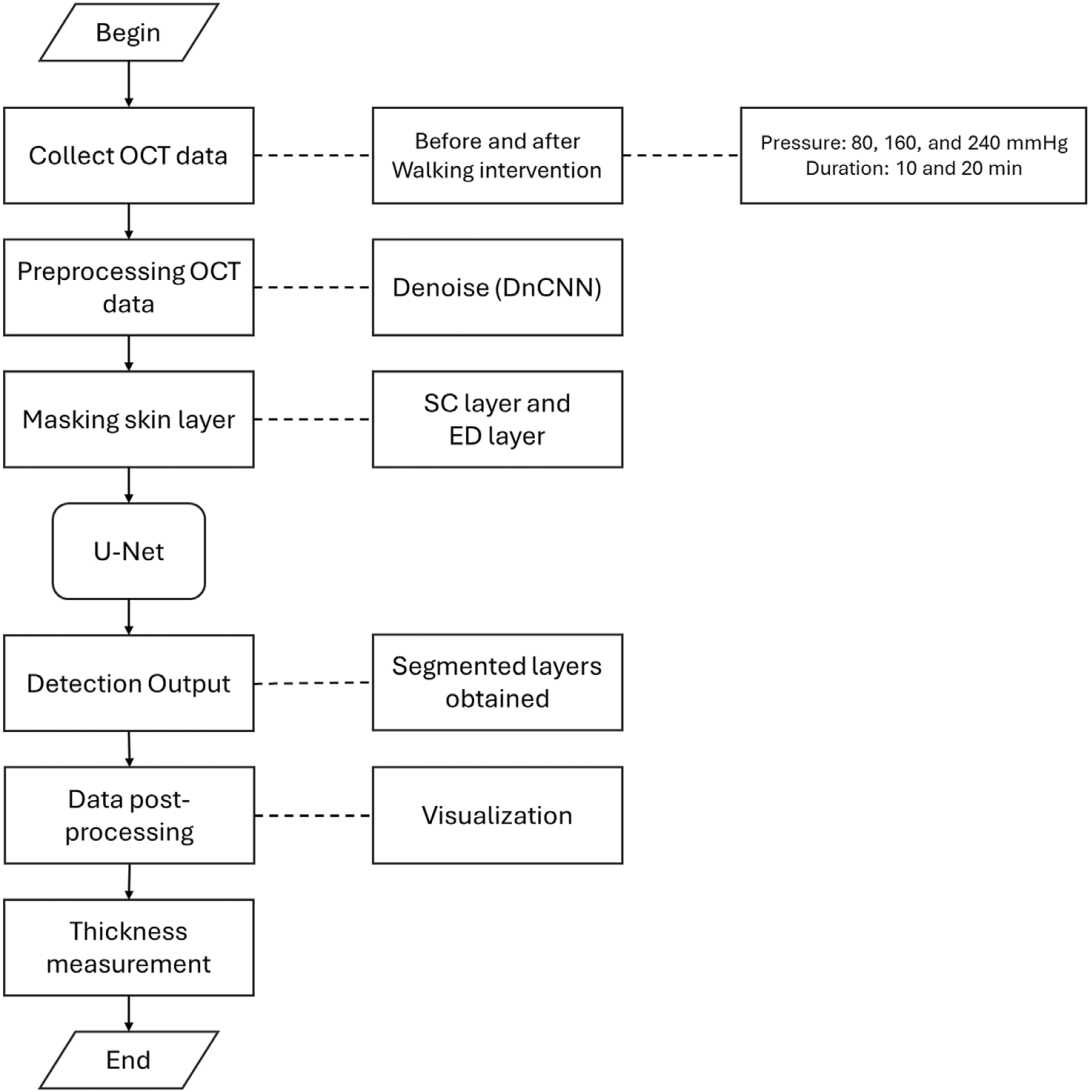
Process flow of data processing for object detection in deep learning.

In our study, the OCT image dataset was pre‐processed before applying the CNN. Each image derived from the 3D bin files from the OCT device was converted to grayscale and cropped into a standardized 997 × 997 pixel square (equal to 5 mm length × 5 mm width × 5 mm depth) to ensure consistency and direct the model's attention to the region of interest [34] (Figure 4). A denoising Convolutional Neural Network (DnCNN) was then used to reduce noise by learning the residual features from clean images. Following pre‐processing, manual masking was performed to isolate the SC and ED areas. This step helps the model focus only on the relevant regions, which is essential in medical imaging to prevent interference from the background or unrelated areas [37].

**FIGURE 5:**
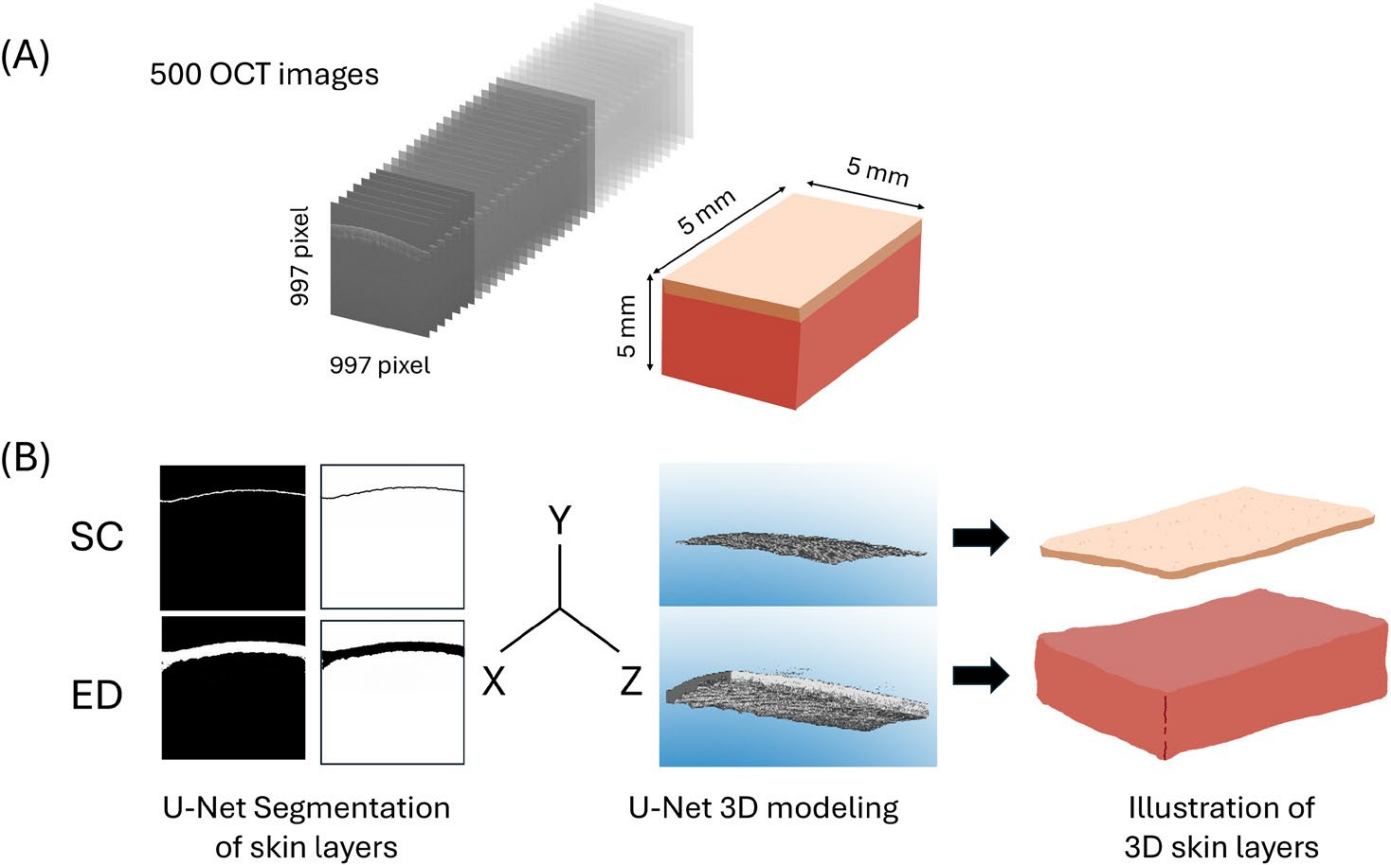
OCT image processing illustration. (A) 500 OCT images to integrate the volume of SC and ED. (B) The 3D view of the *X*, *Y*, and *Z* axes of the SC and ED. ED, living epidermis; SC, stratum corneum.

In this study, the thickness was derived from the ratio of volume to surface area derived from the OCT 3D model, a method that accounts for uneven geometry and yields a more representative average tissue thickness across the region of interest. When volume data are unavailable, angle meters or curvature‐corrected measurement tools may be used to adjust for topographical variation [38]. The *X*‐ and *Y*‐axes are from a single frame of the OCT image, and the *Z*‐axis is constructed from the 500 OCT images, resulting in a 3D model allowing for precise analysis of structural changes and abnormalities (Figure 5A). U‐Net segmentation was used to enable automatic calculation of SC and ED thicknesses. In the initial measurement, the thickness was measured in millimeters (mm) and then converted to μm. The U‐Net architecture is particularly effective in this process because it captures high‐level features and intricate details, making it ideal for segmenting complex skin structures [37]. Segmented SC and ED images are described in the *X*‐, *Y*‐, and *Z*‐axes as *X* is the width, *Y* is the depth, and *Z* is the length (Figure 5B).

### 2.5 Statistical Analysis

The values for the thickness of the SC and ED are presented as mean ± standard error. To evaluate the effects of insole hardness, walking duration, and their interaction on SC and ED thickness, a 3 × 2‐way Analysis of Variance (ANOVA) was conducted. One‐way ANOVA followed by Fisher's least significant difference (LSD) post hoc test was applied to compare the SC and ED thicknesses across the three insole hardnesses (80, 160, and 240 mmHg) within each walking duration (10 and 20 min) [5, 10, 11]. Differences in SC and ED thicknesses between the two walking durations for each insole condition were analyzed using paired *t*‐tests. All statistical tests were performed using a significance threshold of *p* < 0.05. Data analysis was performed using Statistical Package for Social Science (SPSS) version 20 (SPSS Inc., Chicago, IL, USA).

## 3 Result

In this study, we evaluated the interaction between insole hardness and duration using a two‐way ANOVA, then we evaluated SC and ED Thickness using a one‐way ANOVA with a 3 × 2 (insole hardness and duration) factorial design, and further, we evaluated three plantar regions, T1, M1, and M2, of 10 and 20 min using a paired *t*‐test. One‐way ANOVA showed that an insole hardness factor of 80 mmHg caused a significant main effect of SC thickness change in M1 (*p* = 0.041); the interaction of 80 mmHg in 20 min caused a significant main effect of SC thickness change in M1 (*p* = 0.019), and the interaction of 160 mmHg in 20 min caused a significant main effect of SC thickness in T1 (*p* = 0.031) and M1 (*p* = 0.002). However, there was no significant interaction between insole hardness and the duration of SC and ED Thickness. The plantar thickness in the SC and ED was assessed immediately after each walking intervention.

### 3.1 Segmentation Result

In our study, it was found that the U‐Net model performed exceptionally well when segmenting the skin layer. Furthermore, the Dice coefficient of 0.89 and Intersection over Union (IoU) of 0.82 provide further evidence of the robustness of the model. Moreover, the loss function converged efficiently after 50 epochs, stabilizing at 0.045, demonstrating the model's excellent generalization abilities. Accordingly, the accuracy for segmenting the SC reached 92% and the accuracy for segmenting the ED reached 96%, and the total average for both layers' accuracy is 94%.

### 3.2 Effect of Insole Hardness on Walking Duration

A two‐way ANOVA revealed no significant multivariate main effect of air‐insole pressure on plantar skin parameters, Wilks' *Λ* = 0.885, *F*(18,296) = 1.30, *p* = 0.185. Similarly, no significant main effect of walking duration was observed, Wilks' *Λ* = 0.955, *F*(9,148) = 0.77, *p* = 0.648. There was also no significant interaction effect between air‐insole pressure and walking duration, Wilks' *Λ* = 0.919, *F*(18,296) = 0.71, *p* = 0.798. Although no statistically significant effects were observed, small variations across plantar regions were noted during exploratory analysis.

### 3.3 Effect of Insole Hardness

**FIGURE 6:**
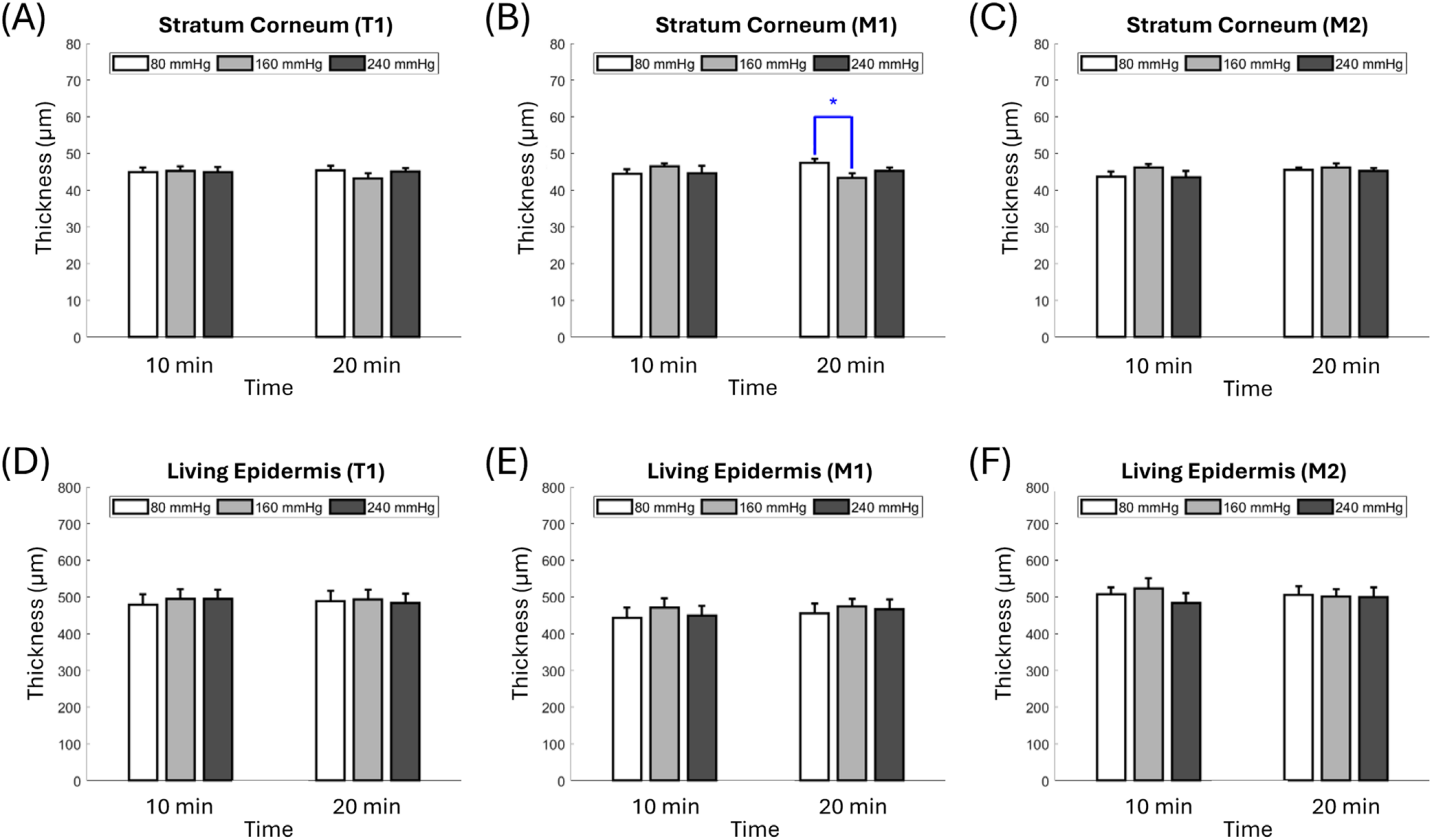
SC and ED thickness; (A) SC 80 mmHg, (B) SC 160 mmHg, (C) SC 240 mmHg, (D) ED 80 mmHg, (E) ED 160 mmHg, (F) ED 240 mmHg. ED, living epidermis; M1, first metatarsal head; M2, second metatarsal head; SC, stratum corneum; T1, big toe; *Significant difference (*p* < 0.05).

In comparing the effects of insole hardness, we found a significant difference between 80 and 160 mmHg in SC thickness in the M1 region after 20 min of walking (ANOVA, *p* < 0.05). No significant differences were observed between the T1 and M2 regions in this comparison. Additionally, there were no significant differences in SC thickness between 80 and 240 mmHg or between 160 and 240 mmHg in any of the regions. For the ED layer, no significant differences were found across all pressure levels (80, 160, and 240 mmHg) and regions (T1, M1, and M2) (Table 1, Figure 6).

**TABLE 1:** ANOVA result on various insole hardness of the thickness in SC and ED layers.

Parameter	Region	Duration	Inner pressure				Fisher LSD		
						One‐way ANOVA *p* value	Post hoc		
			80 mmHg (Mean ± SE)	160 mmHg (Mean ± SE)	240 mmHg (Mean ± SE)		80 mmHg vs. 160 mmHg	80 mmHg vs. 240 mmHg	160 mmHg vs. 240 mmHg
SC thickness	T1	10	44.9 ± 1.3	45.3 ± 1.2	45.0 ± 1.4	0.973	0.825	0.962	0.863
		20	45.4 ± 1.3	43.2 ± 1.5	45.1 ± 1.0	0.425	0.226	0.848	0.306
	M1	10	44.4 ± 1.3	46.5 ± 0.8	44.6 ± 2.0	0.549	0.318	0.907	0.378
		20	47.4 ± 1.1	43.3 ± 1.3	45.3 ± 0.9	0.041*	0.012*	0.190	0.210
	M2	10	43.7 ± 1.4	46.2 ± 0.9	43.6 ± 1.6	0.316	0.204	0.933	0.176
		20	45.5 ± 0.7	46.2 ± 1.1	45.2 ± 0.8	0.717	0.569	0.828	0.432
ED thickness	T1	10	479.5 ± 27.6	495.4 ± 26.4	494.6 ± 26.1	0.893	0.674	0.691	0.982
		20	488.3 ± 28.5	492.6 ± 26.7	483.9 ± 25.0	0.974	0.910	0.908	0.819
	M1	10	443.5 ± 28.3	471.0 ± 25.3	449.0 ± 26.6	0.744	0.470	0.886	0.562
		20	456.4 ± 26.5	474.2 ± 21.1	467.1 ± 26.0	0.876	0.611	0.760	0.838
	M2	10	507.2 ± 19.1	523.8 ± 27.1	484.0 ± 26.5	0.518	0.633	0.507	0.256
		20	505.3 ± 24.3	500.4 ± 21.0	500.1 ± 25.6	0.985	0.884	0.877	0.992

Abbreviations: ANOVA, Analysis of Variance; ED, living epidermis; LSD, least significant difference; M1, first metatarsal head; M2, second metatarsal head; SC, stratum corneum; SE, standard error; T1, big toe.

* Significant difference (*p* < 0.05).

### 3.4 Effect of Walking Duration

**FIGURE 7:**
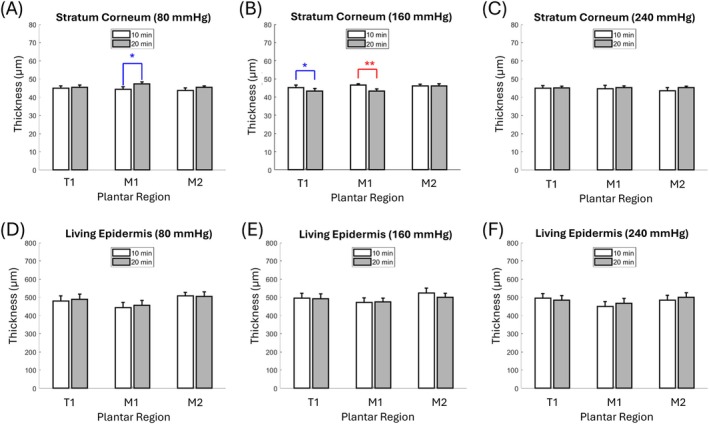
SC and ED thickness; (A) SC 80 mmHg, (B) SC 160 mmHg, (C) SC 240 mmHg, (D) ED 80 mmHg, (E) ED 160 mmHg, (F) ED 240 mmHg. ED, living epidermis; M1, first metatarsal head; M2, second metatarsal head; SC, stratum corneum; T1, big toe. *Significant difference (*p* < 0.05); **Significant difference (*p* < 0.01).

**FIGURE 8:**
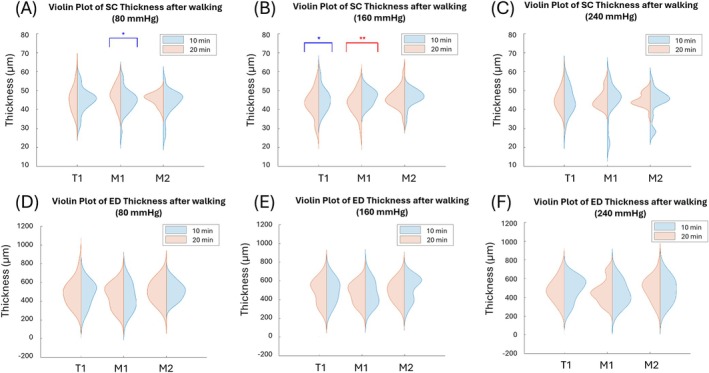
SC and ED thickness violin plot distribution; (A) SC 80 mmHg, (B) SC 160 mmHg, and (C) SC 240 mmHg, (D) ED 80 mmHg, (E) ED 160 mmHg, (F) ED 240 mmHg. ED, living epidermis; M1, first metatarsal head; M2, second metatarsal head; SC, stratum corneum; T1, big toe. *Significant difference (*p* < 0.05); **Significant difference (*p* < 0.01).

Upon comparing the effect of walking durations, we found that the thickness of the SC in the M1 region significantly increased after walking for 20 min with an 80 mmHg insole hardness (*p* < 0.05). Furthermore, we found that the thickness of SC in the T1 and M1 regions significantly decreased after walking for 20 min with a 160 mmHg insole hardness (*p* < 0.05). However, there were no significant differences in the thickness of the SC in M2 for all walking durations and insole hardness. In addition, regarding the thickness of the ED layer, no statistically significant differences were found between walking durations across all pressure levels (80, 160, and 240 mmHg) (Table 2, Figure 7). Furthermore, violin plots were produced to display the distribution of SC and ED thickness over three insole hardnesses and two walking durations in three different plantar regions. These plots display both the median values and the spread of the data, enabling a clearer comparison between conditions (Figure 8).

**TABLE 2:** Pair t‐test result on various walking duration of thickness in SC and ED layers.

Pressure	Thickness	Location	Duration		
			10 min	20 min	Pair *t*‐test *p*‐Value
			Mean ± SE	Mean ± SE	
80 mmHg	SC (μm)	T1	44.9 ± 1.3	45.4 ± 1.3	0.608
		M1	44.4 ± 1.3	47.4 ± 1.1	0.019 *
		M2	43.7 ± 1.4	45.5 ± 0.7	0.250
	ED (μm)	T1	479.5 ± 27.6	488.3 ± 28.5	0.657
		M1	443.5 ± 28.3	456.4 ± 26.5	0.510
		M2	507.2 ± 19.1	505.3 ± 24.3	0.891
160 mmHg	SC (μm)	T1	45.3 ± 1.2	43.2 ± 1.5	0.031 *
		M1	46.5 ± 0.8	43.3 ± 1.3	0.002 **
		M2	46.2 ± 0.9	46.2 ± 1.1	0.999
	ED (μm)	T1	495.4 ± 26.4	492.6 ± 26.7	0.867
		M1	471.0 ± 25.3	474.2 ± 21.1	0.829
		M2	523.8 ± 27.1	500.4 ± 21.0	0.265
240 mmHg	SC (μm)	T1	45.0 ± 1.4	45.1 ± 1.0	0.952
		M1	44.6 ± 2.0	45.3 ± 0.9	0.666
		M2	43.6 ± 1.6	45.2 ± 0.8	0.288
	ED (μm)	T1	494.6 ± 26.1	483.9 ± 25.0	0.526
		M1	449.0 ± 26.6	467.1 ± 26.0	0.187
		M2	484.0 ± 26.5	500.1 ± 25.6	0.295

Abbreviations: ED, living epidermis; M1, first metatarsal head; M2, second metatarsal head; SC, stratum corneum; T1, big toe.

* Significant difference (*p* < 0.05).

** Significant difference (*p* < 0.01).

## 4 Discussion

This study examined the effects of varying insole hardness and walking duration on plantar skin thickness, specifically in the SC and ED layers. Our findings revealed a significant change in M1 after 20 min of walking. Furthermore, we observed a significant increase in SC thickness in the T1 region, using an insole hardness of 80 mmHg in 20 min. We also observed a significant decrease in SC thickness in the T1 and M1 regions using an insole hardness of 160 mmHg in 20 min. No significant differences were observed in the ED thickness of M2, suggesting a region‐specific and pressure‐specific response. In contrast, the ED thickness showed no significant differences under any condition, indicating that the SC thickness may be more sensitive to changes in deformation under different insole hardness and walking duration under mechanical stress.

OCT provides superior resolution for evaluating the fine structure of the skin, particularly the SC and ED layers. OCT provides details of the curved nature of the skin surface, especially in weight‐bearing regions such as the plantar foot, which presents a challenge for accurate thickness measurements. Because the skin is not flat, using direct linear distances may introduce errors owing to slope and curvature artifacts [39]. Accurate thickness estimation is essential for interpreting skin and soft tissue adaptation to mechanical loading, where incorrect assessment may misrepresent the structural integrity of the tissue [40, 41]. Some studies have used high‐frequency sonography (HFUS) in dermatology, which has gained traction; however, the resolution and contrast of ultrasound images have not met expectations [32]. Although HFUS images clearly show the dermis and hypodermis, the epidermis cannot be accurately portrayed [42]. Thus, OCT provides higher resolution and accuracy than ultrasound for detecting SC and ED thickness.

The effect of insole hardness, according to our study, walking using an 80 mmHg insole hardness for 20 min resulted in a significant increase in SC thickness at the M1 compared to the 160 mmHg and 240 mmHg, suggesting a beneficial response in SC tissue hydration and perfusion [5]. One possible explanation for the increase in SC thickness may involve tissue adaptation or microcirculatory responses [43]. Thus, lower insole hardness may contribute to maintaining plantar tissue compliance and reducing stiffness during walking, which may provide insight into plantar loading conditions [10]. The M1 region experiences some of the highest plantar pressures during gait, making it a common site for foot complications, especially in individuals with DM [44]. When the skin over this area is thin and stiff, mechanical stress can lead to excessive friction and callus formation, which further increases plantar pressure beneath the skin [45]. Furthermore, excessive compression over hard insole surfaces can amplify plantar stress, accelerate skin and tissue fatigue, and increase the likelihood of callus formation and ulcer development [18].

According to our study, the effect of walking duration on SC thickness at T1 and M1 at 160 mmHg after 20 min may be due to increased soft tissue compression arising from mechanical stress and transient fluid redistribution [39, 46]. The reduction in T1 and M1 thickness may indicate compromised skin barrier function, as it may be associated with changes in hydration or barrier properties [47]. According to Chao et al., people with DM tend to have thicker epidermis in their feet, but when diabetic neuropathy and DFU develop, the thickness of the epidermis decreases [25]. Therefore, the combination of 160 mmHg and a longer walking duration may have exceeded the adaptive response of the plantar tissue in healthy individuals, potentially contributing to temporary structural changes within the SC and ED layers. This observation may indicate that prolonged mechanical loading may influence tissue hydration and alter the mechanical behavior of the plantar skin, resulting in increased stiffness [48]. Although these findings were observed in healthy participants, no direct physiological measurements were performed. However, the absence of significant changes in the ED suggests that deeper tissue structures may maintain greater stability during short‐term intervention. These results are consistent with prior research showing that walking can induce measurable changes in plantar tissue thickness and biomechanics, particularly under specific loading conditions [49].

Interestingly, no significant changes in SC or ED thickness were observed under the 240 mmHg condition. Although higher insole pressures may theoretically impose greater mechanical stress on plantar tissues, the absence of significant responses may suggest a non‐linear relationship between pressure magnitude and epidermal adaptation [50]. One possible explanation is that plantar tissues may exhibit adaptive or compensatory mechanisms under higher loading conditions, thereby limiting measurable structural changes [10]. Another possible explanation is that OCT‐based thickness measurements may have limited sensitivity in detecting additional structural alterations under higher pressure conditions, particularly due to resolution constraints and boundary‐detection challenges [26]. Further studies incorporating larger sample sizes and additional biomechanical or physiological measurements are needed to clarify these mechanisms.

There are some limitations to this study, as this study involved multiple statistical comparisons across plantar regions, skin layers, insole pressures, and walking durations, which may increase the risk of Type I error. Although exploratory analyses identified several significant findings, no formal correction for multiple comparisons was applied because of the limited sample size and exploratory nature of the study. Therefore, the findings should be interpreted cautiously and require confirmation in larger future studies. Furthermore, because this study only included healthy non‐DM adults, the results should be interpreted carefully when relating them to people with DM, whose plantar tissues may exhibit different structural, metabolic, and biomechanical responses to loading conditions. Moreover, while this study focused on the SC and ED, thickening in these layers may be associated with underlying changes in the dermis, such as increased collagen cross‐linking, fibrosis, or altered extracellular matrix composition, which can reduce tissue elasticity and perfusion and increase mechanical stress during walking [25]. Therefore, investigating the relationship between SC, ED, and dermal remodeling may contribute to a better understanding of plantar tissue responses relevant to DFU prevention research [20]. In addition, although both 10 and 20 min walking interventions were tested, the results indicated that the 20 min duration had a more pronounced effect on changes in plantar tissue thickness at an insole hardness of 80 mmHg. This suggests that tissue responses may be duration‐dependent, and findings from shorter walking periods may not fully capture the extent of mechanical adaptation or stress accumulation [7, 11, 51]. However, a longer loading time in areas with high localized pressure may cause more fluid to be expelled, resulting in thinner and smaller tissues [40]. Future studies with longer walking durations and inclusion of DM populations could provide further insight into how prolonged loading affects plantar tissue behavior.

## 5 Conclusion

Monitoring changes in soft tissue thickness in the SC and ED with OCT, particularly in high‐risk regions such as M1, offers a practical method for assessing tissue response to mechanical stress during walking. As the SC and ED are the outermost and most reactive layers, they serve as an early indication of stress or friction of abnormal plantar loading. We found increased soft tissue thickness in the SC at 80 mmHg with a 20 min walking duration. Thus, our findings suggest that selecting low‐pressure insole hardness can maintain healthier SC and ED layer conditions during exercise, which could support future investigations into plantar tissue management in populations vulnerable to DFU‐related complications.

## Author Contributions

Conceptualization: Yih‐Kuen Jan and Chi‐Wen Lung. Methodology: Ardha Ardea Prisilla, and Gilang Titah Ramadhan. Investigation: Ben‐Yi Liau, Chien‐Cheng Tai, Sheena Christabel Pravin, Kiruthika Venkataramani, Winson Chiu‐Chun Lee, and Congo Tak Shing Ching. Writing – original draft: Ardha Ardea Prisilla. Writing – review and editing: Yih‐Kuen Jan and Chi‐Wen Lung. All authors have read and agreed to the published version of the manuscript.

## Funding

This study was supported by grants from the National Science and Technology Council of Taiwan (NSTC 114‐2923‐E‐468‐001‐MY3 and NSTC 114‐2410‐H‐468‐016). The funding agency was not involved in data collection, analysis, or interpretation.

## Ethics Statement

The studies involving humans were approved by Central Regional Research Ethics Committee China Medical University, Taichung, Taiwan (CRREC‐112‐130). This study was registered in the International Trial Registry [ClinicalTrials.gov: Identifier NCT06746597] with the first posted Study Registration Dates on 2024‐12‐24. The study was conducted in accordance with local legislation and institutional requirements. The participants provided written informed consent to participate in this study.

## Conflicts of Interest

The authors declare no conflicts of interest.

## Data Availability

The authors confirm that the data supporting the findings of this study are available within the article.
